# *In Vitro* Antibacterial and Antibiofilm Activities of Chlorogenic Acid against Clinical Isolates of *Stenotrophomonas maltophilia* including the Trimethoprim/Sulfamethoxazole Resistant Strain

**DOI:** 10.1155/2013/392058

**Published:** 2012-12-20

**Authors:** Arunkumar Karunanidhi, Renjan Thomas, Alex van Belkum, Vasanthakumari Neela

**Affiliations:** ^1^Department of Medical Microbiology and Parasitology, Faculty of Medicine and Health Sciences, Universiti Putra Malaysia, Selangor 43400 Serdang, Malaysia; ^2^La Balme Microbiology Unit, BioMérieux, 3 route de Port Michaud, 38390 La Balme-les-Grottes, France

## Abstract

The *in vitro* antibacterial and antibiofilm activity of chlorogenic acid against clinical isolates of *Stenotrophomonas maltophilia* was investigated through disk diffusion, minimum inhibitory concentration (MIC), minimum bactericidal concentration (MBC), time-kill and biofilm assays. A total of 9 clinical *S. maltophilia* isolates including one isolate resistant to trimethoprim/sulfamethoxazole (TMP/SMX) were tested. The inhibition zone sizes for the isolates ranged from 17 to 29 mm, while the MIC and MBC values ranged from 8 to 16 **μ**g mL^−1^ and 16 to 32 **μ**g mL^−1^. Chlorogenic acid appeared to be strongly bactericidal at 4x MIC, with a 2-log reduction in viable bacteria at 10 h. *In vitro* antibiofilm testing showed a 4-fold reduction in biofilm viability at 4x MIC compared to 1x MIC values (0.085 < 0.397 A 490 nm) of chlorogenic acid. The data from this study support the notion that the chlorogenic acid has promising *in vitro* antibacterial and antibiofilm activities against *S. maltophilia*.

## 1. Introduction

*Stenotrophomonas maltophilia* is an emerging nosocomial pathogen. Although it is an organism of a limited pathogenicity, *S. maltophilia* infections has been associated with high morbidity and mortality especially among immunocompromised individuals. *S. maltophilia, *which is a high producer of biofilm, frequently colonizes patient secretions (respiratory, urine, and wound exudates), fluids (intravenous fluids), catheters (central venous), and implants used in the hospital setting. It is associated with a wide range of infections including bacteraemia, endocarditis, meningitis, respiratory tract, and skin and soft tissue infections [1]. Community acquired *S. maltophilia* infections have also been reported especially among patients with cystic fibrosis [2, 3]. *S. maltophilia *is intrinsically resistant to several classes of antibiotics. Nosocomial *S. maltophilia* strains are multiple drug resistant (MDR) and are usually less susceptible to carbapenems, cephalosporins, penicillins, chloramphenicol, and fluoroquinolones which severely complicates the treatment options. Currently, the combination drug, trimethoprim/sulfamethoxazole (TMP/SMX), remains as the main therapy of choice worldwide [1]. But, growing resistance to TMP/SMX has become a serious threat in the treatment of *S. maltophilia* infections. In addition, problems associated with contraindication of TMP/SMX therapy in patients due to toxicity and intolerance, spread of acquired mobile resistance determinants, and reduced susceptibilities to available antibiotics [4, 5] prompt for an expanded search of new antibacterial agents. 

Chlorogenic acid is a polyphenolic natural compound which is commonly present in plant materials such as apples, coffee beans, grapes, pulp, peel, and tea leaves [6]. Structurally, it is an ester of caffeic acid with the 3-hydroxyl group of a quinic acid. It has been reported to possess many health benefits including antibacterial, antifungal, antiviral, antiphlogistic, antioxidant, chemopreventive, and other biological activities [7–12]. Extensive *in vitro* antimicrobial activities and possible action mode have been reported on a variety of Gram-positive and Gram-negative bacterial pathogens [13].

Classical antibiotics target a specific reaction, whereas natural antimicrobial compounds like plant polyphenols inhibit several different groups of biomolecules in a pathogen. Therefore, the development of resistance to such compounds is unlikely, which makes them as an attractive antibacterial candidate [14, 15]. In addition, synthetic antibiotics are more focused on planktonic cells than biofilms, while natural antibiotic compounds are proven to inhibit bacterial adhesion and biofilm development [16]. Since there is a great demand for natural substances that inhibit bacterial planktonic cells as well as biofilms, we investigated the *in vitro* antibacterial and antibiofilm efficacy of chlorogenic acid against a collection of clinical *S. maltophilia* isolates.

## 2. Materials and Methods

### 2.1. Bacterial Isolates and Culture Conditions

Nine clinical isolates of *S. maltophilia* including one TMP/SMX resistant isolate (MIC > 32 *μ*g mL^−1^) as per CLSI guidelines [17] were obtained from the Microbiology Unit at Hospital Kuala Lumpur (HKL), Malaysia. The source of the isolates included cerebrospinal fluid (CSF, *n* = 1), tracheal aspirate (*n* = 4), bronchoalveolar lavage (*n* = 1), pus (*n* = 2), and urine (*n* = 1). Bacterial samples were cultured onto nutrient agar slants and transported to the Medical Microbiology Laboratory at the Faculty of Medicine and Health Sciences, Universiti Putra Malaysia (UPM). The isolates were presumptively identified as *S. maltophilia* by Gram stain, catalase, oxidase, alpha hemolysis on blood agar, fermentation on MacConkey agar, DN*ase* production, and mannitol fermentation on mVIA agar medium. The isolates were further genotypically confirmed as *S. maltophilia *by single stranded PCR (SS-PCR) [18, 19]. Confirmed isolates were stored in Luria-Bertani (LB) broth (Merck Pvt. Ltd., Selangor, Malaysia) supplemented with 20% glycerol at −80°C. Multiple copies of working cultures stored at −20°C were thawed in ice and used for further experiments. No culture stocks (−20°C) were used more than two times as repeated passaging may lose some of the bacterial properties. 

### 2.2. Chemicals

Chlorogenic acid, tetrazolium (XTT) and menadione were purchased from Sigma Aldrich (St. Louis, MO, USA). Dimethyl sulfoxide (DMSO) and bacterial growth media such as Mueller-Hinton agar (MHA), Mueller-Hinton broth (MHB), Luria-Bertani (LB) broth, and Brain Heart Infusion broth (BHI) were purchased from Merck Pvt. Ltd., Selangor, Malaysia. Stock solution (64 mg mL^−1^) of chlorogenic acid was prepared in 10% DMSO (Merck Pvt. Ltd., Selangor, Malaysia) and different concentrations of the test antibacterial material were prepared by serial dilution ranging from 32 to 0.25 mg mL^−1^. Serially diluted chlorogenic acid was stored in sterile eppendorf tubes awaiting further investigation. 

### 2.3. Disk Diffusion Assay

The antibacterial activity of chlorogenic acid against *S. maltophilia* was assessed by the disk diffusion method [20]. Isolates were grown in LB broth to a turbidity of 0.5 McFarland standards and, using sterile cotton swabs, the bacterial inoculum was spread onto MHA plates by lawn culture. Sterile antibiotic assay filter paper discs (Whatman, GE Healthcare, Malaysia) of 6 mm diameter were placed on MHA plates and 20 *μ*L of chlorogenic acid from each sets ranging from 0.25 to 32 mg mL^−1^ (0.25, 0.5, 1, 2, 4, 8, 16, and 32) was carefully loaded onto the filter paper discs. TMP/SMX antibiotic disc (Oxoid Limited, Hampshire, UK) was included as an antibiotic control, while paper disc impregnated with 20 *μ*L of DMSO (10%) was used as a negative control. *S. maltophilia* ATCC 13637 was included as a quality control. All plates were incubated at 37°C for 24 h until visible growth was observed and the inhibition zones formed around the filter papers discs were measured and compared with the control antibiotic. The experiment was performed in triplicates.

### 2.4. Susceptibility Testing

The minimum inhibitory concentration (MIC) of chlorogenic acid for *S. maltophilia* was determined by broth dilution method as described previously by Schwalbe et al. [21]. For MIC testing, chlorogenic acid at a concentration of 1024 *μ*g mL^−1^ was prepared from the previously prepared stock solution. Briefly, 11 sterile screw capped test tubes (10 mm × 100 mm) 1 through 11 were labeled and arranged in a rack. Tubes 2–11 were loaded with 500 *μ*L of sterile MHB and 500 *μ*L of chlorogenic acid from the freshly prepared stock solution (1024 *μ*g mL^−1^) was pipetted into tubes 1 and 2. Twofold serial dilution was performed by transferring 500 *μ*L of the mixture from tube 2 to tube 3 and successive transference was continued through tube 9. To avoid carryover of antibiotic, pipette tips were changed between each tube. From tube 9, 500 *μ*L of the solution was discarded. Tube 10 was used as a growth control and received no antibiotic. Except the 11th tube (broth control), all tubes were inoculated with 500 *μ*L of bacterial inoculum (10^6^ CFU mL^−1^) and incubated at 37°C for 24 h. After incubation, the lowest concentration of the antibiotic at which no visible growth occurred was considered as MIC. Minimum bactericidal concentration (MBC) was determined by spread plating 100 *μ*L of the broth from clear wells onto MHA plates followed by incubation at 37°C for 24 h. The lowest concentration of an antimicrobial agent at which all the cells were killed is defined as MBC. The experiment was performed in triplicates.

### 2.5. Time-Kill Assay for Detecting the Bactericidal Effect of Chlorogenic Acid

The killing kinetics of chlorogenic acid at 0x, 1/2x, 1x, 2x, and 4x MIC values was determined by the method described previously [22] with minor modifications. Different concentrations of chlorogenic acid were added to each tube containing 4.5 mL of MHB. The tubes were inoculated with 5 × 10^5^ colony forming unit (CFU mL^−1^) of *S. maltophilia* ATCC 13637 strain grown in BHI broth (Merck Pvt. Ltd., Selangor, Malaysia) and incubated at 37°C in an orbital shaker, set at 130 rpm. Tube without chlorogenic acid served as growth control (0x). An aliquot of 100 *μ*L was collected at 0 min, 2.5 h, 5 h, 10 h, and 24 h postinoculation. Tenfold serial dilution of broth culture was plated onto MHA plates and the CFU mL^−1^ of each sample was determined by a spread plate technique incubated upon further incubation for 24 h. The experiment was performed in duplicates.

### 2.6. Effect of Chlorogenic Acid on ****S. maltophilia**** Performed Biofilms

In order to test the efficacy of chlorogenic acid on biofilm, *S. maltophilia* ATCC 13637 was subjected to biofilm formation by the method described by Peeters et al. [23] with the following modifications. The test strain was grown in BHI broth and when the cells reached to a turbidity of 0.5 McFarland standard, cell suspensions were added to 48 wells of a flat-bottomed polystyrene 96-well polystyrene microtiter plate (Orange Scientific, Belgium). To ensure proper adhesion of bacterial cells to the polystyrene surface, the plate was incubated at 37°C for 24 h at static conditions. Following 24 h of adhesion and biofilm formation, planktonic cells were removed from the wells and the plate was rinsed with 100 *μ*L of phosphate buffered saline (PBS). Cells adhered to the polystyrene microtitre plate were treated with 100 *μ*L of chlorogenic acid at 1x, 2x, and 4x MICs. For biofilm experiment, 24 wells of a microtiter plate inoculated with 100 *μ*L of these dilutions were considered as “treated” and 24 control wells without the test compound were considered as “nontreated.” Nontreated cells were incubated with 100 *μ*L of DMSO (10%) which served as a negative control. The plate was further incubated for 12 h at 37°C with gentle shaking and the quantification of biofilm was carried out by XTT calorimetric assay as mentioned below. The experiment was repeated two times in order to achieve reproducibility.

### 2.7. Biofilm Quantification by XTT Assay

A modified XTT assay [23] was carried out to quantify the *S. maltophilia* performed biofilms on polystyrene microtitre plates. Briefly, 0.4 mg of XTT in 1 mL PBS was mixed with 10 *μ*L of menadione in 10 mL acetone (Merck Pvt. Ltd., Selangor, Malaysia). The contents were mixed thoroughly and 100 *μ*L of the XTT/menadione solution was added to all wells followed by incubation in the dark at 37°C for 5 h. The contents of the wells were microfuged at 15,000 g for 4 min and 100 *μ*L of clear supernatant from each well was transferred to a fresh 96-well flat-bottomed microtiter plate before the absorbance was read at 490 nm using (BioTek EL808, USA) a microplate reader.

**Statistics**

Statistical analysis was performed using SPSS version 16.0 software 2007 (SPSS Inc., Chicago, IL). All assays were performed at least in duplicates. For biofilm inactivation studies, mean values between treated and untreated samples were tested for significance by Student's *t*-test. The significant difference in biofilm reduction at different concentrations of chlorogenic acid was compared with the control (strain without the test compound was normalized as 100%). The significant level was set at *P* < 0.05.

## 3. Results

### 3.1. Characterization of Isolates

All the isolates were confirmed as *S. maltophilia* by phenotypic and genotypic methods. Eight isolates were susceptible to TMP/SMX by disk diffusion, whilst one isolate (isolate number 4) from CSF was found to be TMP/SMX resistant (MIC > 32 *μ*g mL^−1^) and interpreted according to CLSI guidelines. 

### 3.2. Disk Diffusion Assay

**Figure 1:**
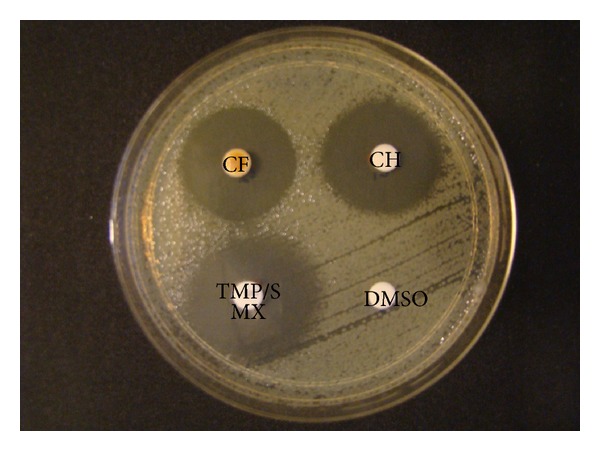
Effect of chlorogenic acid (32 mg mL^−1^) applied to a blank filter paper disk on MHA plate inoculated with *S. maltophilia* ATCC 13637. Caffeic acid which is an ester of chlorogenic acid also showed similar zone size. CF:caffeic acid, CH:chlorogenic acid, TMP/SMX:trimethoprim/sulfamethoxazole (30 *μ*g), and DMSO:dimethyl sulfoxide (10%).

The results obtained from disk diffusion test are illustrated in Table 1. The zone of inhibition ranged between 17–29 mm at 32 mg mL^−1^ of chlorogenic acid and a consistent increase in zone sizes were observed at increasing concentrations of the chlorogenic acid. The maximum activity was observed at 32 mg mL^−1^ with a zone size of 29 mm. The compound was highly active against 3 isolates (*n* = 4, 30, and 49) and a representative antibacterial pattern of chlorogenic acid against *S. maltophilia* ATCC 13637 is shown in Figure 1. Chlorogenic acid was found to be antibacterial against all the clinical isolates including the TMP/SMX resistant strain.

### 3.3. MIC and MBC Determination

The results of the MIC indicated that chlorogenic acid inhibited all the bacterial isolates tested and the respective MIC and MBC values of chlorogenic acid for *S. maltophilia* were presented in Table 1. The MICs were in the range of 8 to 16 *μ*g mL^−1^ in which 7 isolates were inhibited at 8 *μ*g mL^−1^ and 2 isolates were inhibited at 16 *μ*g mL^−1^. Isolate number 4 being TMP/SMX resistant was also susceptible to chlorogenic acid at 16 *μ*g mL^−1^. Meanwhile, the MBCs of chlorogenic acid for *S. maltophilia* ranged between 16–32 *μ*g mL^−1^.

### 3.4. Analysis of Bacterial Killing Kinetics

**Figure 2:**
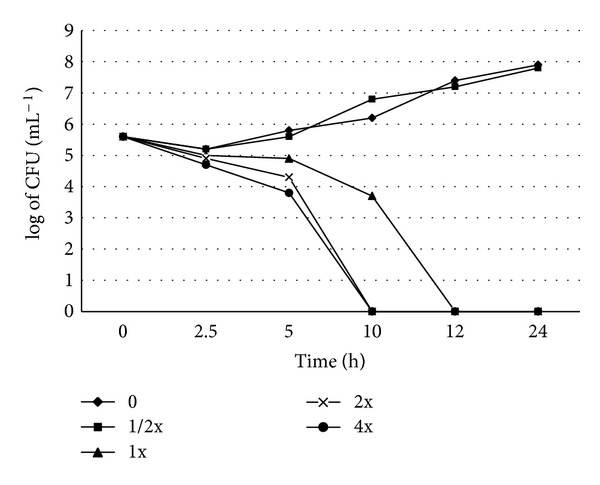
Effect of chlorogenic acid on the viability of *S. maltophilia* ATCC 13637 in liquid medium (time-kill curve). *S. maltophilia* ATCC 13637 grown at 37°C in the presence of chlorogenic acid at concentrations of 0 (♦), 4 (■), 8 (▲), 16 (×), and 32 (●) *μ*g mL^−1^ (1/2x, 1x, 2x and 4x MIC) with control (0 MIC). MIC: minimal inhibitory concentration; CFU: colony-forming units.

Time-kill assay indicated that *S. maltophilia* ATCC 13637 cells grown in MHB maintained their viability for at least 24 h of incubation, whereas *S. maltophilia* treated with chlorogenic acid at increasing concentrations (8, 16, and 32 *μ*g mL^−1^) killed more than 90% of the cells and no viable cells could be enumerated after 12 h of incubation. Twofold and four fold MICs of chlorogenic acid inhibited the growth in 10 h demonstrating a dose-dependent killing property of chlorogenic acid (Figure 2). The bactericidal endpoint for *S. maltophilia* at 4x and 1x MIC was achieved at 10 h and 12 h time points, respectively, with an approximate reduction of CFU by 2 log units (99.9%). 

### 3.5. Biofilm Inhibition with Chlorogenic Acid

**Figure 3:**
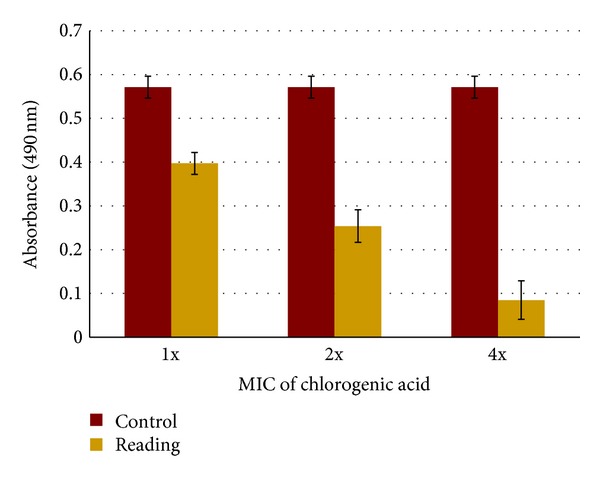
Effect of chlorogenic acid on biofilm viability of *S. maltophilia* ATCC 13637 at concentrations of 8, 16, and 32 *μ*g mL^−1^ (1x, 2x, and 4x MIC) with control (0 MIC). Comparison of absorbance between control and treated samples at 490 nm by XTT assay. XTT, 2,3-*bis*(2-methoxy-4-nitro-5-sulfo-phenyl)-2H-tetrazolium-5-carboxanilide; MIC: minimal inhibitory concentration.

Challenging biofilms of *S. maltophilia* ATCC 13637 with chlorogenic acid at increasing concentrations of MIC showed significant reduction in biofilm viability. The effect of chlorogenic acid tested at 1x, 2x, and 4x MICs on the viability of established biofilms (Figure 3) was generally dose dependent. Compared to 1x MIC (8 *μ*g mL^−1^), chlorogenic acid at 32 *μ*g mL^−1^ (4x MIC) exhibited greater activity against *S. maltophilia* biofilms with a 4-fold reduction (0.085< 0.397 A 490 nm) in biofilm viability.

## 4. Discussion

There exists a global threat for the existing antimicrobial agents due to the widespread emergence of microbial drug resistance. The discovery and development of new antibiotics to fight the “superbugs” still remain a great challenge for researchers. Since the emergence of bacterial drug resistance remains at full pace, novel antimicrobial agents to counter antibiotic-resistant organisms have great demands in the market [24]. Opportunistic pathogens like *S. maltophilia* are frequently encountered in hospitalized patients implanted with indwelling medical devices like catheters, prosthetic heart valves, contact lenses, and so forth [25–27]. Both clinical and environmental isolates of *S. maltophilia* are capable of adhering to biotic and abiotic surfaces such as glass, plastic materials, polyvinyl chloride, and Teflon [28, 29]. This study is one of the first reports demonstrating the antibacterial and antibiofilm activities against *S. maltophilia*. Our results demonstrate that chlorogenic acid is very active against *S. maltophilia*, including the TMP/SMX resistant strain (MIC > 32 *μ*g mL^−1^). Previously, chlorogenic acidand chlorogenic acid containing plant materials have been shown to have antiviral [30], antifungal [11, 31], and strong antibacterial activities. Several Gram-positive (*Staphylococcus aureus, Streptococcus pneumoniae*, *Streptococcus mutans *and *Bacillus subtilis*) [13, 16, 19, 31] and Gram-negative (*Salmonella typhimurium*,* Shigella dysenteriae*, and *Escherichia coli*) [13] bacterial pathogens have been reported to be highly susceptible to chlorogenic acid. At a concentration of 32 *μ*g mL^−1^, chlorogenic acid was able to produce larger zones on MHA plates ranging from 17 to 29 mm. The zone sizes are comparable with the control antibiotic TMP/SMX (30 *μ*g). Compared to the TMP/SMX susceptible isolates, chlorogenic acid was highly active against the TMP/SMX resistant strain isolated from CSF.

The compound has greater solubility in DMSO which makes it more permeable as well as promoting its bactericidal property. Pure chlorogenic acid is effective in killing *S. maltophilia* clinical isolates with MICs ranging between 8–16 *μ*g mL^−1^. The antibiotic resistant strain was inhibited at 16 *μ*g mL^−1^. The compound also extended its antibacterial activity against other pathogens like *Acinetobacter iwoffii*, methicillin resistant *S. aureus,* and *Pseudomonas aeruginosa* determined by disk diffusion test in our earlier investigation of chlorogenic acid against an array of bacterial pathogens (data not shown). A recent study by Lou et al. [13] has shown that chlorogenic acid has broad spectrum antibacterial activity with MIC ranging from 20 to 40 *μ*g mL^−1^ against Gram-positive and 20 to 80 *μ*g mL^−1^ against Gram-negative organisms. However, this is the first report on a natural compound against *S. maltophilia* with low MIC values which is slightly less than other gram-negative organisms reported. Although the precise mechanism of action of chlorogenic acid is not completely understood, a recent study by Li et al. have suggested that chlorogenic acid or chlorogenic acid related derivatives from plants could possibly inhibit certain enzymes involved in the bacterial fatty acid synthesis, such as FabI and FabG [22]. This supports the notion that chlorogenic acid being a polyphenolic compound has strong inhibitory effects against *S. maltophilia*. Meanwhile, chlorogenic acid also significantly increased the permeability of outer membrane and plasma membrane in *S. dysenteriae and S. pneumoniae *which resulted in the leakage of cytoplasmic contents including nucleotides [13].

Previous *in vitro* antimicrobial studies using chlorogenic acid [13, 16, 19, 31] have reported the antimicrobial potential of the compound, but none has investigated the bactericidal activity of chlorogenic acid against emerging pathogens like *S. maltophilia*. Knowledge on killing kinetics of chlorogenic acid will be highly useful in the mechanism of action, transcription analysis, and gene expression studies. In *S. maltophilia*, killing was observed at a lower concentration of chlorogenic acid due to its lower MIC (8–16 *μ*g mL^−1^). A concentration dependent killing was observed in case of *S. maltophilia*, where a 2x and 4x MICs exhibited strong bactericidal activities in 10 h. At subinhibitory concentration (0.5x MIC), a slight decrease in the colony count was observed initially. However, bactericidal activity was not detected and there was an evidence of regrowth after 5, 10, and 24 h of incubation. A rapid decrease in colony count was observed between 5–10 h of incubation and complete killing was achieved after 10 h at 2x and 4x MICs. At 1x MIC, the killing time of chlorogenic acid was at 12 h. These findings were partly in agreement with the observations made earlier by Lou et al. [13] who reported the bactericidal activity at 12 h when tested with ethyl acetate fraction of burdock leaves containing chlorogenic acid on food related Gram-positive and Gram-negative pathogens. 

Very few studies have been reported on the effect of chlorogenic acid on biofilm formation. Against *S. maltophilia* biofilms, chlorogenic acid showed bactericidal activity. A recent study by Stauder et al. [16] showed that coffee high molecular weight (cHMW) melanoidin compounds containing chlorogenic acid as one of the active components were able to abolish *S. mutans* (major causative agent of human dental caries) biofilms *in vitro*. In this study, we observed a significant 4-fold reduction in biofilm viability at 4x MIC concentration (32 *μ*g mL^−1^) of chlorogenic acid. *S. maltophilia* is capable of attaching to polystyrene wells in less than 2 h of incubation; however, maximum biofilm growth endpoint on the surface of polystyrene wells occurs at 24 h [32]. Although, biofilm experiment remains time consuming because of the slow growing property of *S. maltophilia*, the microtiter well plate method and XTT assay were found to be suitable, reproducible, and quantitative methods. To the best of our knowledge, no data exist concerning natural product activity against *S. maltophilia* biofilms. In the present study, for the first time, we tested the effects of different concentrations of chlorogenic acid on *S. maltophilia* performed biofilms. Our results showed that chlorogenic acid at 1x, 2x, and 4x MICs significantly reduced the adhesion of *S. maltophilia* to polystyrene in a dose-dependent manner. These findings could be of interest because *S. maltophilia* is resistant to commonly available broad spectrum antibiotics, including *β*-lactams, aminoglycosides, and quinolones [3]. 

## 5. Conclusions

In conclusion, the findings of this study highlight the antibacterial and antibiofilm properties of chlorogenic acid against emerging nosocomial pathogen *S. maltophilia*. Complete eradication of microbial biofilms still remains a crucial step and a great challenge for clinicians and researchers. Superbugs like *S. maltophilia *are strong biofilm producers with intrinsic resistance to many antibiotics. Natural antimicrobial agents like chlorogenic acid which is ecologically safe and less hazardous than synthetic bactericides displayed promising antistenotrophomonas activity on planktonic as well as biofilm forms of *S. maltophilia*. Therefore, chlorogenic acid could be used as a safe alternative to synthetic antimicrobial drugs or in antimicrobial combinations against *S. maltophilia *infections. 

## Figures and Tables

**Table 1:** Zone of inhibitions, MICs, and MBCs of chlorogenic acid against *S. maltophilia*.

Isolate number	Zone of inhibition in diameter (mm)^a^				
	20 *μ*L of chlorogenic acid (32 *μ*g mL^−1^)	TMP/SMX^b^ (30 *μ*g)	DMSO (10%)	MIC (*μ*g mL^−1^)^c^	MBC (*μ*g mL^−1^)^d^
4	29	0	—	16	32
16	20	32	—	8	32
24	17	31	—	8	32
26	18	31	—	8	32
30	27	32	—	8	16
40	19	32	—	8	32
49	26	32	—	8	16
51	24	32	—	16	32
53	25	32	—	8	32
ATCC 13637	27	31	—	8	16

^a^Determined by disk diffusion assay.

^b^Trimethoprim/sulfamethoxazole.

^c^Determined by macrobroth dilution method.

^d^Determined by plate colony count technique.

—: No zone of inhibition.
